# The Emerging Role of Rhinoviruses in Lower Respiratory Tract Infections in Children – Clinical and Molecular Epidemiological Study From Croatia, 2017–2019

**DOI:** 10.3389/fmicb.2019.02737

**Published:** 2019-12-03

**Authors:** Sunčanica Ljubin-Sternak, Tomislav Meštrović, Irena Ivković-Jureković, Branko Kolarić, Anamarija Slović, Dubravko Forčić, Tatjana Tot, Maja Mijač, Jasmina Vraneš

**Affiliations:** ^1^Molecular Microbiology Department, Dr. Andrija Štampar Teaching Institute of Public Health, Zagreb, Croatia; ^2^Medical Microbiology Department, School of Medicine, University of Zagreb, Zagreb, Croatia; ^3^Clinical Microbiology and Parasitology Unit, Polyclinic “Dr. Zora Profozić”, Zagreb, Croatia; ^4^University Centre Varaždin, University North, Varaždin, Croatia; ^5^Department of Pulmonology, Allergy, Immunology and Rheumatology, Children’s Hospital Zagreb, Zagreb, Croatia; ^6^Faculty for Dental Medicine and Healthcare/School of Medicine, Josip Juraj Strossmayer University of Osijek, Osijek, Croatia; ^7^Department of Epidemiology, Dr. Andrija Štampar Teaching Institute of Public Health, Zagreb, Croatia; ^8^Faculty of Medicine, University of Rijeka, Rijeka, Croatia; ^9^Center of Excellence for Virus Immunology and Vaccines, Center for Research and Knowledge Transfer in Biotechnology, University of Zagreb, Zagreb, Croatia; ^10^Department of Microbiology, General Hospital Karlovac, Karlovac, Croatia

**Keywords:** rhinovirus, species, epidemiology, phylogenetic analysis, children, lower respiratory tract infection

## Abstract

Rhinoviruses (RVs) are increasingly implicated not only in mild upper respiratory tract infections, but also in more severe lower respiratory tract infections; however, little is known about species diversity and viral epidemiology of RVs among the infected children. Therefore, we investigated the rhinovirus (RV) infection prevalence over a 2-year period, compared it with prevalence patterns of other common respiratory viruses, and explored clinical and molecular epidemiology of RV infections among 590 children hospitalized with acute respiratory infection in north-western and central parts of Croatia. For respiratory virus detection, nasopharyngeal and pharyngeal flocked swabs were taken from each patient and subsequently analyzed with multiplex RT-PCR. To determine the RV species in a subset of positive children, 5′UTR in RV-positive samples has been sequenced. Nucleotide sequences of referent RV strains were retrieved by searching the database with Basic Local Alignment Tool, and used to construct alignments and phylogenetic trees using MAFFT multiple sequence alignment tool and the maximum likelihood method, respectively. In our study population RV was the most frequently detected virus, diagnosed in 197 patients (33.4%), of which 60.4% was detected as a monoinfection. Median age of RV-infected children was 2.25 years, and more than half of children infected with RV (55.8%) presented with lower respiratory tract infections. Most RV cases were detected from September to December, and all three species co-circulated during the analyzed period (2017–2019). Sequence analysis based on 5′UTR region yielded 69 distinct strains; the most prevalent was RV-C (47.4%) followed by RV-A (44.7%) and RV-B (7.9%). Most of RV-A sequences formed a distinct phylogenetic group; only strains RI/HR409-18 (along with a reference strain MF978777) clustered with RV-C strains. Strains belonging to the group C were the most diverse (41.6% identity among strains), while group B was the most conserved (71.5% identity among strains). Despite such differences in strain groups (hitherto undescribed in Croatia), clinical presentation of infected children was rather similar. Our results are consistent with newer studies that investigated the etiology of acute respiratory infections, especially those focused on children with lower respiratory tract infections, where RVs should always be considered as potentially serious pathogens.

## Introduction

Rhinoviruses (RVs) are small, non-enveloped viruses that belong to the family *Picornaviridae*, genus *Enterovirus*. To date, there are 171 rhinovirus (RV) genotypes recognized and classified into the three species as RV-A (83 types), RV-B (32 types), and RV-C (56 types) (Royston and Tapparel, 2016; Pan et al., 2018). RV-A and RV-B were discovered by isolation on monkey kidney cells in 1950s (Price, 1956) while RV-C genotypes, which are not cultivable using ordinary culture methods, have been identified decades after following the rise of molecular techniques (Lamson et al., 2006; Lau et al., 2007). There are also differences between RV species in utilization of cell entry receptor: a majority of RV-A and RV-B attach to the intercellular adhesion molecule (ICAM)-1 (classified as the major receptor group) and the others alternatively bind low density lipoprotein receptor (LDL-R) (minor receptor group), whereas RV-C utilizes human cadherin-related family member 3 (CDHR3) (Bochkov et al., 2015; Royston and Tapparel, 2016).

Rhinovirus genome is a 7.2-kb single-stranded, positive-sense RNA with a single open reading frame (ORF) joined to a 5′ untranslated region (5′UTR) and a short viral priming protein (VPg) (Jacobs et al., 2013). ORF encodes a poly-protein which is cleaved by virally encoded proteases in 11 proteins. Four proteins – VP1, VP2, VP3, and VP4 – make up the viral capsid and account for the virus’ antigenic diversity, while the remaining non-structural proteins are involved in viral genome replication and assembly. A rather conserved 5′ UTR region that harbors internal ribosomal entry site (IRES) is usually utilized for RV detection from clinical samples, while more precise genotyping is based on VP4/VP2 or VP1 sequence analysis.

Rhinovirus have been neglected for decades, primarily because they were considered less virulent and only capable of causing mild common cold, and were not recognized, until recently, as important causative agents of lower respiratory tract infections (LRTIs) and severe respiratory disease (To et al., 2017). Introduction of sensitive and technically simple molecular detection assays, especially multiplex PCR, enabled affordable RV detection in line with other common respiratory viruses in clinical samples. Together with coronaviruses, RVs are indeed responsible for majority of upper respiratory tract infection (URTI), but also for substantial rate of LRTIs in all age groups (Lauinger et al., 2013; Zhao et al., 2018, Čivljak et al., 2019). Many studies showed that RV is one of the leading causes of pneumonia, bronchiolitis and other form of severe respiratory disease (Zhao et al., 2018), standing side by side with respiratory syncytial virus (RSV) in children and influenza in elderly (Esposito et al., 2013; Ning et al., 2017; Čivljak et al., 2019). There are several studies that report increased rates of asthma exacerbations and LRTIs among children with RV-C when compared to those infected with RV-A and RV-B (Bizzintino et al., 2011; Lauinger et al., 2013); however, recent studies did not find any relationship between a specific RV species and the severity of clinical presentation (Jacobs et al., 2015; van der Linden et al., 2016; Zhao et al., 2018).

Data on RV prevalence in Croatia are scarce, and molecular epidemiology was thus far not described. This study aims to determine the RV prevalence, compare it with prevalence patterns of other common respiratory viruses, as well as to explore clinical and molecular epidemiological features of RV infections among hospitalized children with acute respiratory infection.

## Materials and Methods

### Ethical and Safety Issues

The research was performed in accordance with relevant guidelines/regulations and in line with the Declaration of Helsinki, as revised in 2013. Written informed consent was obtained from all participants (children’s parents or their legal guardians). The study was approved by the Ethics Committee of the Dr. Andrija Štampar Teaching Institute of Public Health and conducted as part of the Croatian Science Foundation project entitled “New and neglected respiratory viruses in vulnerable groups of patients” (No. IP-2016-06-7556). All standard biosecurity and institutional safety procedures have been adhered to.

### Patients and Sample Collection

From May 2017 to April 2019, a total of 590 patients were included from hospitals located in north-western and central part of Croatia: Clinical Hospital Zagreb and General Hospital Karlovac, respectively. Inclusion criteria were: age lower than 18 years, a clinical diagnosis of acute respiratory tract infection (ARI), and need for hospitalization [on ward (≥1 day) or day hospital (for more than 6 but less than 24 h)]. Exclusion criteria were presumed bacterial respiratory infection – including otitis, sinusitis and bacterial pneumonia, healthcare-associated infection, and ambulatory treated patients.

Patients were categorized into the four groups according to their respective age (i.e., <1, 1–2.99, 3–4.99 and ≥5 years of age), and two groups according to the localization of infection in those with upper respiratory tract infection (URTI) and lower respiratory tract infection (LRTI). URTI was defined by symptoms of the common cold, coryza, cough, and hoarseness with or without fever, so clinical syndromes of respiratory catarrh, rhinitis and/or pharyngitis represented URTI category. LRTI was defined by symptoms of tachypnea, wheezing, severe cough, breathlessness, and by specific clinical signs such as nasal flaring, jugular and intercostal retractions, cyanosis (in rare instances), as well as wheezing, crackles and inspiratory rhonchi or generally reduced breath sounds during auscultation. Clinical syndromes of bronchitis, bronchiolitis and pneumonia were included in LRTI category (Tregoning and Schwarze, 2010; Ljubin-Sternak et al., 2016). To avoid unnecessary X-ray exposure, chest radiographs were taken only for some of the patients in order to exclude or confirm bacterial pneumonia.

For respiratory virus detection, nasopharyngeal and pharyngeal flocked swabs from each patient were collected, combined, and placed in viral transport medium (UTM^TM^, Copan, Italy). Specimens were immediately transported to the Molecular microbiology laboratory at the Public Health Institute where they were stored at −80°C until tested. The results of virology testing were released to the physicians periodically, approximately once per week. As a part of routine care, nasopharyngeal, pharyngeal swabs, and blood cultures were taken from hospitalized patients and submitted for bacterial diagnostics using standard cultivation methods. Demographic and clinical data, antimicrobial use records, and the results of routine bacterial studies were collected by a retrospective review of patient charts.

### Multiplex RT-PCR

To isolate viral DNA and RNA from viral transport medium, 300 μL has been extracted according to the manufacturer’s protocol using Ribospin^TM^ vRD Kit (GeneAll Biotechnology, Seoul, Korea). Multiplex RT-PCR for 15 respiratory viruses using Seeplex^®^ RV15 detection kit (Seegene Inc., Seoul, Korea) was performed. Briefly, multiplex PCR and cDNA synthesis as one-step reaction was performed and set up in three different tubes with three sets of primers. More specifically, “A set” contained primers for simultaneous amplification of target sequences of adenovirus (AdV), human coronavirus (HCoV) 229E/Nl63, parainfluenza virus (PIV) types 1-3, and PCR internal control to check for the presence of substances that may interfere with amplification; “B set” contained primers for HCoV OC43, RV groups A/B/C, RSV type A and B, influenza (Flu) type A, and PCR internal control; and “C set” contained primers for human bocavirus (HBoV), Flu type B, human metapneumovirus (HMPV), PIV type 4, human enterovirus (HEV), and the overall process control (human RNase P was included throughout the entire process as a control from nucleic acid extraction to amplification). Amplification was performed on thermal cycler GeneAmp^®^ 9700 PCR System (Applied Biosystems, Foster City, United States). Detection of PCR products was done by microchip electrophoresis on the MCE^®^-202 MultiNA device (Shimadzu, Kyoto, Japan) – including software analysis showing results in the form of electropherograms and virtual image gels.

### Rhinovirus Typing – Reverse Transcription, PCR and Sequencing

To determine RV species, 5′ UTR of the genome in RV-positive samples was sequenced. Total RNA was extracted from 500 μL of RV-positive samples by the method reported by Chomczynski and Mackey (1998). Reverse transcription was performed at 42°C for 60 min, in a reaction mix containing 10 μL of isolated RNA, 1 × PCR buffer (GE Healthcare, United Kingdom), 0.1 mM of each dNTP, 20 U of RNase inhibitor (Thermo Fisher Scientific, United States), 1.25 mM MgCl2, 2.5 mM of random hexanucleotide primers and 50 U of MuLV reverse transcriptase (Thermo Fisher Scientific, United States) in a final volume of 20 μL.

PCR reaction was performed by amplifying 5′ UTR, using NTR + (5′ CAA GYA CTT CTG TYT CCC 3′) and NTR- (5′ CAC GGA CAC CCA AAG TAG T 3′) primer pair, modified from Wisdom et al. (2009), corresponding to positions 161–178 and 531–549 of strain A2 (acc. no. X02316.1), respectively. Final PCR mixtures contained 1 × OneTaq PCR buffer (New England Biolabs, United States), 10 mM dNTP, 0.25 mM MgCl2, 0.25 mM of each primer, and 1.25 U of OneTaq DNA polymerase (New England Biolabs, United States). The amplified products were separated on a 1.5% agarose gel, excised and purified by centrifugation through glass wool (Sun et al., 2012).

Sequencing reactions were set up with purified DNA, one of the specific primers used for amplification, and a BigDye Terminator v3.1 Cycle Sequencing Kit (Thermo Fisher Scientific, United States) according to the manufacturer’s protocol. Sequencing and sequence analysis were performed on a 3130 Genetic Analyzer (Thermo Fisher Scientific, United States).

### Phylogenetic Analysis

Nucleotide sequences of referent RV strains were retrieved by searching the database with BLAST (Basic Local Alignment Tool)^1^ and used to construct alignments and phylogenetic trees. Alignments were performed using MAFFT multiple sequence alignment tool available at the EMBL-EBI website^2^, and edited in AliView v1.23 (Larsson, 2014). Phylogenetic trees were generated using the maximum likelihood method with Molecular Evolutionary Genetics Analyses (MEGA) software v6.06 (Tamura et al., 2013), under the most appropriate model of nt substitution determined with jModeltest v2.1.4 (Darriba et al., 2012). Bootstrap probabilities for 1,000 iterations were calculated to evaluate confidence estimates. Sequence conservation (defined as percentage of genomic positions identical in all strains; gaps were ignored during computing) and evolutionary distances (*p*-distances) within and between groups have been calculated using MEGA 6.06.

The sequences of HRSV strains obtained in this study were deposited in the GenBank under acc. nos. MN369460 – MN369528.

### Statistical Analysis

Data analysis was performed using Stata/MP (Ver.15.1; StataCorp LLC, College Station, United States). Age was presented using medians with stated interquartile range (IQR). Demographic and clinical parameters were compared by *χ*^2^-test or Fisher’s exact test for categorical variables and by Kruskal–Wallis test for continuous variables. To assess the strength of association between dependent variable (RV positive patients) and age we used univariate logistic regression. *P*-value was set to <0.05.

## Results

### Prevalence of Respiratory Viruses in Patient Groups

In total, 590 patients were screened for respiratory viruses. The male:female ratio was 346:244 (1.42:1). The median age was 1.75 years (range 7 days to 17 years). According to the age groups, there were 210 (35.6%) patients in the group <1 years of age, 168 (28.5%) patients from 1-2.99 years of age, 68 (11.5%) patients from 3−4.99 years, and 144 (24.4%) patients with ≥5 years of age. Clinical diagnosis of URTI has been determined for 277 (46.9%) patients, and LRTI for 313 (53.1%) patients. Any type of respiratory virus has been detected in 451 (76.4%) patients; in 315 (69.8%) of positive patients there was a monoinfection with a single virus, while in 136 (30.2%) positive patients two or more viruses were detected concurrently.

The exact prevalence pattern of detected viruses can be seen in Table 1. RV was the most frequently detected virus, diagnosed in 197 patients (33.4%); 60.4% as monoinfection, and 39.6% as coinfection with other respiratory viruses. This was followed by RSV (19.3%), AdV (15.6%), PIVs (9.5%), Flu types A and B (7.6%), HCoV 229/NL63 and OC43 (7.1%), HBoV (5.3), HEV (4.6%), and HMPV (3.1%).

**TABLE 1:** Prevalence of respiratory viruses detected by multiplex PCR in the study population.

**Virus**	**Number of detections (monoinfection +coinfection)**	**Prevalence (%)**	**95% CI**
Rhinoviruses	197 (119 + 78)	33.4	29.6−37.4
Respiratory syncytial virus	114 (66 + 48)	19.3	16.2−22.7
Adenoviruses	92 (26 + 66)	15.6	12.8−18.8
Parainfluenza type 1–4	56 (33 + 23)	9.5	7.2−12.1
Influenza type A and B	45 (35 + 10)	7.6	5.6−10.1
Coronaviruses 229E/NL63 and OC43	42 (16 + 26)	7.1	5.2−9.5
Bocavirus	31 (6 + 25)	5.3	3.6−7.4
Enterovirus	27 (5 + 22)	4.6	3.0−6.6
Human metapneumovirus	18 (11 + 7)	3.1	1.8−4.8

### Respiratory Viruses According to Age and Anatomic Localization of Infection

**FIGURE 1:**
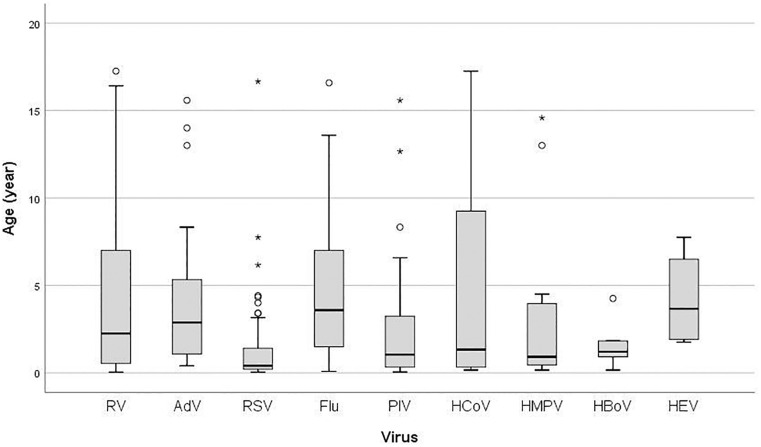
Box plot of patient’s age infected with: RV, rhinovirus; AdV, adenovirus; RSV, respiratory syncytial virus; Flu, Influenza virus type A and B; PIV, parainfluenza virus type 1–4; HCoV, coronavirus 229E/NL63 and OC43; HMPV, human metapneumovirus; HBoV, human bocavirus; HEV, human enterovirus; the ends of the box are the upper and lower quartiles, the median is marked by a vertical line inside the box and the ends of the whiskers represent the minimum and maximum of all of the data; data not included between the whiskers are plotted as an outlier with a dot.

There was a significant difference in age according to the specific virus (*P* < 0.001) (Figure 1). Median age in years of RV infected children (2.25, IQR 6.50) was higher than in children infected with RSV (0.41, IQR 1.29), PIVs (1.04, IQR 3.00), HCoV (1.33, IQR 9.16), HMPV (0.92, IQR 4.09), and HBoV (1.21, IQR 1.71), but lower than in those infected with Flu (3.58, IQR 5.59), AdV (2.88, IQR 4.44), and HEV (3.66, IQR 5.29) (Figure 1). There was no statistically significant difference in the prevalence of RV infection between age groups (*P* = 0.781). Additionally, age (1 year increase) was not significant predictor for RV positivity (OR = 1.01, 95% CI = 0.97–1.06; *P* = 0.534).

**FIGURE 2:**
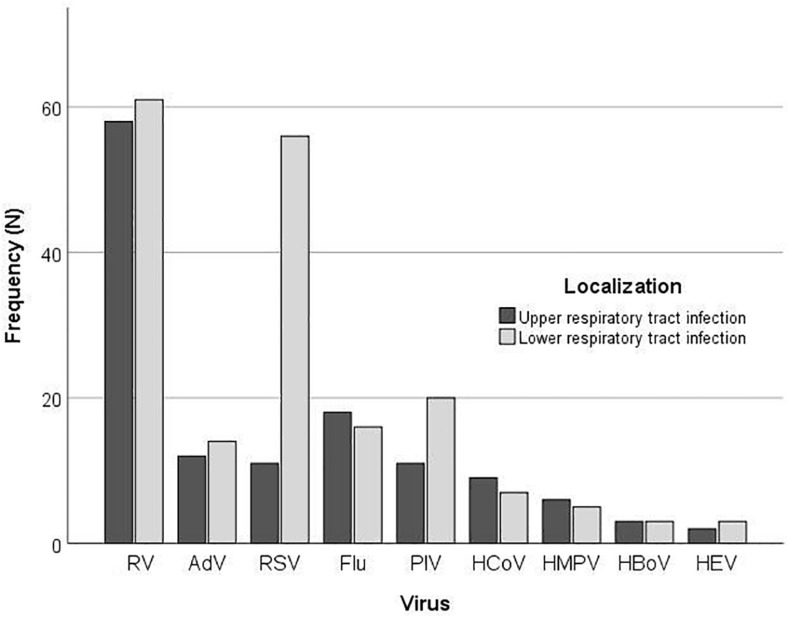
Frequency of virus detection by multiplex PCR according to the localization of infection.

According to the clinical presentation, there was a significant difference in the proportion of LRTIs between the type of the respiratory viruses (*P* = 0.002) (Figure 2). More than half of children infected with RV (110; 55.8%) presented with LRTI; nonetheless, this was not significantly different from the proportion of RV positive children with URTI (87; 44.2%) (*P* = 0.336), while children with RSV infection significantly more often presented with LRTI (*P* < 0.001).

### Results of RV Sequence Analysis

**FIGURE 3:**
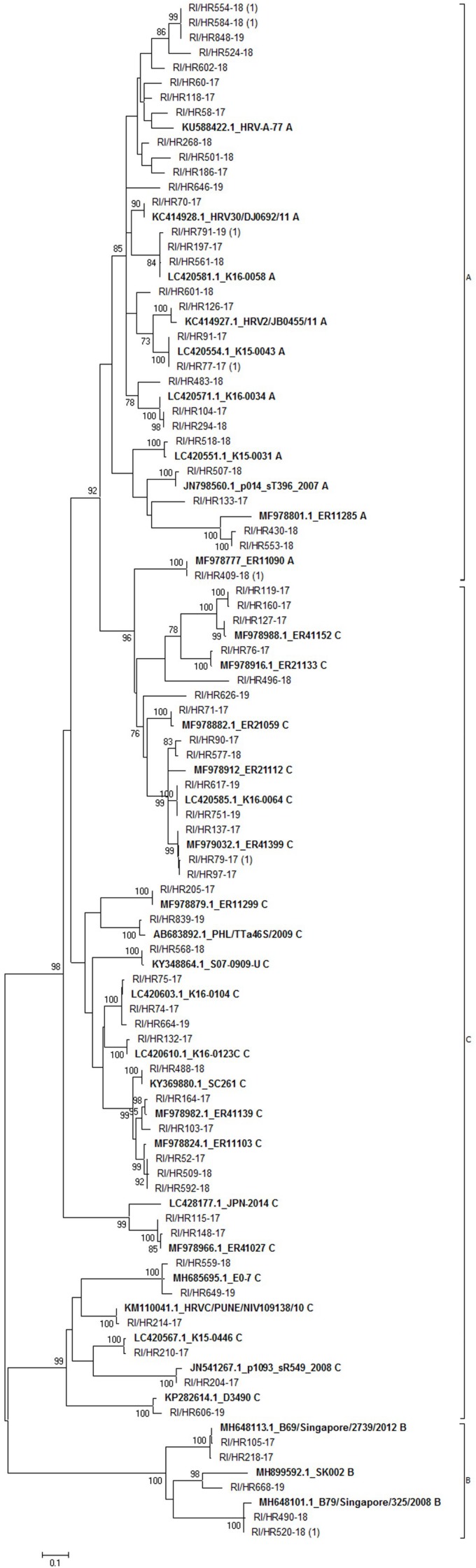
Phylogenetic tree of rhinovirus strains. The tree includes 69 sequences obtained in this study and 34 sequences retrieved from the GenBank. Tree was generated using the maximum-likelihood method; a Hasegawa-Kishono-Yano model with gamma distribution rate and aproportion of invariable sites was implemented. The scale bar indicates the proportion of nucleotide substitutions; the numbers are bootstrap values determined for 1,000 iterations (only values above 70% are shown). Reference strains retrieved from GenBank are shown in bold, their accession numbers, as well as strain name are denoted. Croatian strains have the prefix RI/HR followed by the strain identification number. The numbers in brackets indicate the number of strains with identical sequence.

Out of the 197 RV-positive samples by multiplex PCR, we were able to sequence 76 samples (39%). Sequence analysis based on 395 bp of 5’ UTR region of 76 samples yielded 69 viral types (seven strains had identical sequence) (Figure 3). The most prevalent was RV-C (36/76; 47.4%) followed by RV-A (34/76; 44.7%) and RV-B (6/76; 7.9%). Most of RV-A sequences formed a distinct phylogenetic group; only strain RI/HR409-18 (along with a reference strain MF978777) clustered with RV-C strains (Figure 3).

Of the three respective groups, strains belonging to the group C were the most diverse, with identity of 41.6% (142 of 341 identical positions), while group B was the most conserved with 71.5% identity among strains (241/337). Group A comprised strains which shared an overall 54.3% identical positions (183/337). Calculated p-distances between groups showed group A and C are more closely related (p-distance 0.234) than to group B. Similar *p*-value was calculated between group B and group A (*p*-distance 0.31) or group C strains (p-distance 0.33), respectively.

**FIGURE 4:**
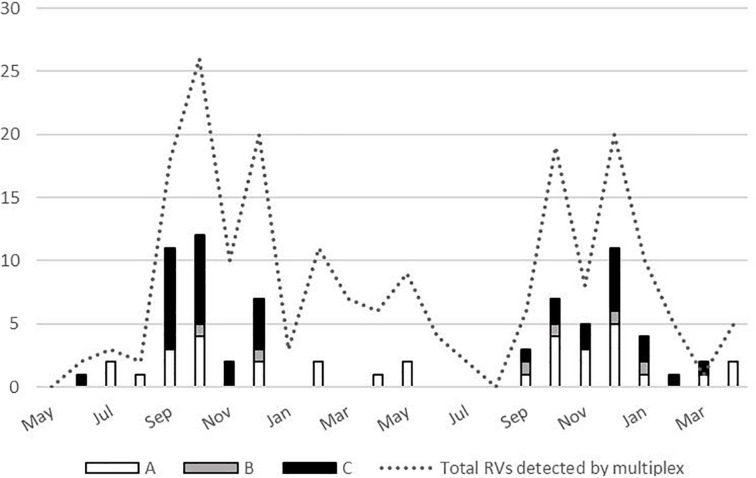
Temporal distribution of the rhinoviruses (RVs) (total *N* = 197, and by species *N* = 76) detected between May 2017 and April 2019.

No significant difference has been demonstrated in clinical symptoms based on the RV species, with the exception of increased frequency of antibiotic treatment in those infected with RV-A species (*P* = 0.012) (Table 2). Most RV cases were detected from September to December, and all three species co-circulated during the analyzed period (Figure 4).

**TABLE 2:** Clinical characteristics of children infected with rhinovirus (RV) according to species.

	**RV-A (*N* = 34)**	**RV-B (*N* = 6)**	**RV-C (*N* = 36)**	**RV-untyped (*N* = 121)**	***P-*value^b^**
Fever in°C^a^ (min-max)	37.3(36.5−39.4)	37(36.4−39.5)	36.9(36.3−39.5)	37(36.1−40.5)	0.162
Hospital stay/days^a^ (min-max)	5.5(2−28)	4(0−7)	5(0−24)	5(0−19)	0.177
Lower respiratory tract infection clinical diagnosis	22(64.7%)	1(16.7%)	21(58.3%)	66(54.5%)	0.091
Bacterial coinfection^c^	2(5.9%)	0(0%)	1(2.9%)	8(6.7%)	0.700
Antibiotic therapy	17(50.0%)	0(0%)	9(25.0%)	51(42.5%)	**0.012**
Oxygen therapy	8(23.5%)	1(16.7%)	8(22.2%)	29(24.2%)	>0.999
ARDS^d^	1(2.9%)	0(0%)	1(2.8%)	7(5.8%9	>0.999
Mechanic ventilation	0(0%)	0(0%)	0(0%)	3(2.5%)	NA^e^

*^*a*^median; ^*b*^Refers to the difference between the three species of viruses, untyped viruses were excluded from the analysis; ^*c*^Three patients had group A or C *Streptococcus* detected in throat swab, three had *Escherichia coli* detected in urine, two patient had *Moraxella catarrhalis* in nasopharyngeal aspirate, one had *E. coli* in tracheal aspirate and one had *Klebsiella pneumoniae* ESBL detected in blood culture; ^*d*^acute respiratory distress syndrome; ^*e*^not applicable.*

## Discussion

In this study we initially evaluated the prevalence of RV and other common respiratory viruses, as well as RV species distribution in hospitalized children with symptoms of ARI over a period of 2 years. Our results are consistent with previously published studies that investigated the etiology of ARI, especially those focused on children with LRTIs (Chen et al., 2015; Ning et al., 2017). Indeed, RV is right next to RSV when addressing the most common causes of bronchiolitis in hospitalized children, as demonstrated in very low-birth-weight infants from Argentina and in one multicentre prospective study from the United States, respectively (Mansbach et al., 2012; Miller et al., 2012).

Although RV can be found both in upper and lower respiratory tract, the potential for spread and the pathophysiology of infection in those two regions differs (as evidenced by studies conducted on RSV) (Kim et al., 2016; González-Parra and Dobrovolny, 2019). Possible causes of such disparity are fundamental differences in the immune responses and virus-cell interactions between these two anatomical regions, resulting in altered disease manifestation and spread (González-Parra and Dobrovolny, 2019). There is also evidence that mucus velocity is decreased in small children (akin to elderly individuals) (Grubb et al., 2016), making them more susceptible to LRTI and creating in turn a potential niche for more detrimental effect of RV infection.

When age is concerned, our study has showed that RV holds a middle ground, not affecting very young children as is the case with RSV. This is comparable with the results found in the study by Cebey-López et al. (2015), where RSV infection was also seen in the youngest age groups (less than 1 year of age), RV was present in somewhat older children (mean between 14.4 and 40.9 months), while AdV predominated in patients older than 50 months. Conversely, some other author groups pointed out how RV can predominate in practically all age groups (Tsagarakis et al., 2018).

In order to determine the RV species, we decided to sequence and analyze 5′ UTR, which has been established as a relatively simple and rapid technique for identifying the RV serotypes in clinical samples. Moreover, it has been shown that 5′ UTR RT-PCR demonstrated greater sensitivity than VP4-VP2 PCR, as reflected by the higher positivity rate in amplification of clinical isolates, and further, no need for a nested PCR or multiple primer pairs – reducing in turn the contamination rate, cost and turnaround time (Kiang et al., 2008). Despite using primers which target the region widely used for typing purposes, more than half of the samples produced no amplicons suitable for sequencing. Such low rate of successful RV sequencing from clinical samples may be a consequence of mutations in primer regions or low viral load in original sample, but most probably arises due to RNA degradation during freezing/thawing cycles.

Phylogenetic groups were readily distinguished and all three RV groups were detected, with group C and A predominating. The proportion of the three RV species revealed in this study (RV-A 44.7%, RV-B 7.9%, and RV-C, 47.4%) is consistent with prior studies worldwide (RV-A, 35.9–67.7%; RV-B, 1.5–13%; RV-C, 23–59.3%) (Lauinger et al., 2013; Rahamat-Langendoen et al., 2013; Jacobs et al., 2015; Tsatsral et al., 2015; Ratnamohan et al., 2016; van der Linden et al., 2016; Zhao et al., 2018).

Intermixing of groups was not observed, except for a single group A sequence (RI/HR409-18) which clustered with group C sequences, indicating a recombination event or co-infection as was proposed by Richter et al. (2015). Furthermore, group B strains were detected sporadically during the analyzed period, which is in accordance with other studies (Lauinger et al., 2013; Launes et al., 2015; Richter et al., 2015). These results represent the first report on RV diversity in Croatia.

In previous reports RV-C (and in lesser extent RV-A) have been associated with more severe illness (Bizzintino et al., 2011; Lauinger et al., 2013; Linder et al., 2013; Chen et al., 2015); however, more recent studies failed to report the connections between species and disease severity (Rahamat-Langendoen et al., 2013; Jacobs et al., 2015; van der Linden et al., 2016; Zhao et al., 2018). In this study there were no significant differences observed in clinical symptoms among three species, except more frequent utilization of antibiotic therapy in patients with RV-A species which can be result of subjective clinical assessment of more severe disease and empirical introduction of therapy.

RV circulated throughout the 2-year period covered by this study with peaks in autumn and winter months. Previous studies reported that RV infections occur all year round, with peaks of infection usually in spring and autumn months (Čivljak et al., 2019). However, recent research endeavors also report peaks in autumn and winter months (Zhao et al., 2018), and some of them specifically note that RV-C demonstrate peak in winter months (Linder et al., 2013). Our study also observed no RV-C detection in spring and summer season.

There are several limitations of the study. Due to cross-sectional study approach, a control group of asymptomatic patients could not have been included (which would facilitate assessment of RV infection severity). Furthermore, we performed only 5′ UTR targeted RT-PCR assay and did not confirm our results with VP4/VP2 or VP1 sequences analysis. Some authors report discordance between proposed phylogeny groups when sequences from the 5’UTR and VP4/VP2 coding regions were analyzed, which can result in imprecise classification (Ratnamohan et al., 2016). More specifically, samples that clustered as RV A using 5′ UTR analysis can be revealed as RV-C when VP4/VP2 region is analyzed (Ratnamohan et al., 2016). Also, complicated rhinovirus infections were excluded from the study. Notwithstanding the aforementioned limitations, this is the first study endeavor cataloging a circulation of RV species in Croatia over 2 years.

## Conclusion

In conclusion, in our study more than half of children infected with RV presented with LRTI, which (together with other newer studies in the field) underlines the need for paradigm shift where RVs will not be merely associated with URTI, but also considered in cases of LRTI. Regardless of the diversity of RV found in this study and the purported heterogeneity of the RV strains infecting the children, the similarity of clinical presentation negates the notion that certain RV species might be more virulent, at least in our case. More clinical and epidemiological studies are warranted to further elucidate this issue, with inevitable use of animal models to study pathogenic specificities of RV infection.

## Data Availability Statement

The datasets generated for this study can be found in the GenBank–accession numbers from MN369460 to MN369528.

## Ethics Statement

The studies involving human participants were reviewed and approved by the Ethics Committee of the Dr. Andrija Štampar Teaching Institute of Public Health and conducted as part of the Croatian Science Foundation project entitled “New and neglected respiratory viruses in vulnerable groups of patients” (No. IP-2016-06-7556). Written informed consent to participate in this study was provided by the participants’ legal guardian/next of kin.

## Author Contributions

SLJ-S, II-J, and JV designed the research. SLJ-S, AS, and DF performed the experiments. MM and TT collected the data. BK, AS, and DF analyzed the data. SLJ-S, TM, MM, TT, II-J, AS, and DF interpreted the data and prepared the draft of the manuscript. JV and TM critically reviewed the draft. SLJ-S and TM wrote the final version of the manuscript. All authors reviewed and approved the final version of the manuscript.

## Conflict of Interest

The authors declare that the research was conducted in the absence of any commercial or financial relationships that could be construed as a potential conflict of interest.

## Footnotes

1 https://blast.ncbi.nlm.nih.gov/

2 http://www.ebi.ac.uk/Tools/msa/mafft
